# Lesion segmentation in breast ultrasound images using the optimized marked watershed method

**DOI:** 10.1186/s12938-021-00891-7

**Published:** 2021-06-07

**Authors:** Xiaoyan Shen, He Ma, Ruibo Liu, Hong Li, Jiachuan He, Xinran Wu

**Affiliations:** 1 College of Medicine and Biological Information Engineering, Northeastern University, Shenyang, China; 2 Key Laboratory of Intelligent Computing in Medical Image, Ministry of Education, Shenyang, China; 3 Department of radiology, Liaoning Cancer Hospital, Shenyang, China

**Keywords:** Breast cancer, Morphological snake, Side window, Ultrasound, Watershed

## Abstract

**Background:**

Breast cancer is one of the most serious diseases threatening women’s health. Early screening based on ultrasound can help to detect and treat tumours in the early stage. However, due to the lack of radiologists with professional skills, ultrasound-based breast cancer screening has not been widely used in rural areas. Computer-aided diagnosis (CAD) technology can effectively alleviate this problem. Since breast ultrasound (BUS) images have low resolution and speckle noise, lesion segmentation, which is an important step in CAD systems, is challenging.

**Results:**

Two datasets were used for evaluation. Dataset A comprises 500 BUS images from local hospitals, while dataset B comprises 205 open-source BUS images. The experimental results show that the proposed method outperformed its related classic segmentation methods and the state-of-the-art deep learning model RDAU-NET. Its accuracy (Acc), Dice similarity coefficient (DSC) and Jaccard index (JI) reached 96.25%, 78.4% and 65.34% on dataset A, and its Acc, DSC and sensitivity reached 97.96%, 86.25% and 88.79% on dataset B, respectively.

**Conclusions:**

We proposed an adaptive morphological snake based on marked watershed (AMSMW) algorithm for BUS image segmentation. It was proven to be robust, efficient and effective. In addition, it was found to be more sensitive to malignant lesions than benign lesions.

**Methods:**

The proposed method consists of two steps. In the first step, contrast limited adaptive histogram equalization (CLAHE) and a side window filter (SWF) are used to preprocess BUS images. Lesion contours can be effectively highlighted, and the influence of noise can be eliminated to a great extent. In the second step, we propose adaptive morphological snake (AMS). It can adjust the working parameters adaptively according to the size of the lesion. Its segmentation results are combined with those of the morphological method. Then, we determine the marked area and obtain candidate contours with a marked watershed (MW). Finally, the best lesion contour is chosen by the maximum average radial derivative (ARD).

## Background

According to the 2020 global cancer data report, breast cancer ranks first among the three most common cancers in women, indicating that it has become a serious threat to the health of women worldwide [1]. Studies show that early detection and diagnosis of breast cancer can effectively increase the cure rate [2]. At present, digital mammography (DM) and breast ultrasound are the two main tools used in breast screening in China. However, ultrasound has no ionizing radiation and can show the anatomy and pathology of dense breast tissue, which DM cannot achieve. Therefore, ultrasound is more suitable for detecting breast lesions in Asian women with high density than DM and is becoming a popular screening tool for breast cancer [3]. Geisel et al. also demonstrated the effectiveness, practicability and feasibility of breast ultrasound as a screening tool for the early detection of occult breast cancer [4]. However, in the process of breast ultrasound imaging, the speckle noise generated by coherent waves greatly reduces the image quality, which requires a high degree of professionalism for radiologists to address. Due to the lack of radiologists in remote areas, ultrasound-based breast cancer screening cannot truly be popularized.

With the development of artificial intelligence technology, computer-aided diagnosis (CAD) systems based on medical images have made great achievements in cancer detection. In particular, the development of an ultrasound-based breast cancer CAD system is impressive. It can realize intelligent screening and diagnosis. When the system receives real-time images, it can perform lesion detection, segmentation and diagnosis automatically. Its application will greatly alleviate the lack of radiologists. However, due to the inherent problems of breast ultrasound (BUS) images such as speckle noise and low contrast, the accuracy of lesion segmentation has not been effectively improved, which greatly affects the reliability of the diagnosis results. Thus, finding a stable and effective BUS image segmentation method is of great significance to promote the application and popularization of ultrasound-based breast cancer CAD systems.

Therefore, we conducted this research. Focusing on solving the inherent problems of BUS images quality, we are committed to designing a stable and efficient image segmentation method. In recent years, many excellent image segmentation algorithms have emerged. Level set, first introduced in 1994 [5] and improved in 1995 [6], 2005 [7], 2012 [8] and so on, has proven to be very effective in image segmentation. However, it needs a great deal of time to solve partial deferential equations (PDEs), which is not very practical. To solve this problem, morphological snake (MS) was proposed. It uses morphological operations on a binary level set to approach the differential operators of a standard PDE [9]. It needs only numerical calculations, so MS is simple and fast. In terms of the field of BUS image segmentation, many scholars have used parameter deformable models and geometric deformable model technology [8, 10, 11]. However, to achieve ideal segmentation results, an appropriate initial tumour boundary or a precise edge-based stop function should be set in advance. Other researchers have used and improved graph-based segmentation methods, such as [12] and [13]. Boukerroui attempted to overcome the biggest drawbacks of the MRF model, i.e., a low optimization speed and local optimization [14]. In 2013, Zhao proposed the generalized fuzzy cluster method (FCM) with spatial information, which performs well in segmentation and has a rapid convergence speed [15]. FCM and the improved FCM algorithm were applied to lesion detection in BUS images [16, 17]. Since 2013, there have been an increasing number of segmentation methods with supervised and semi-supervised learning, especially with the increasing popularity of deep learning, which has made great progress in solving the problem of BUS image segmentation. Supervised learning includes support vector machines, artificial neural networks (ANNs), and convolutional neural networks (CNNs), which have been applied to BUS image segmentation and have made great progress [18–21]. Zhuang proposed the RDAU-NET model [22], which performs best on BUS image segmentation compared to other models. To date, deep learning models have proven to be the best way to perform image segmentation. However, they face some major problems, which are also the main bottleneck for further development. For example, the prediction result is not sufficiently robust. Robustness is the basic performance metric determining whether a model can be widely used [23–26]. Additionally, the model is not explainable, and training data are not sufficient. To solve these problems, a new approach has integrated visual saliency into a deep learning model for BUS image segmentation [27]. Attention blocks were introduced into a U-Net architecture, which learns feature representations that prioritize spatial regions with high saliency levels and achieved a Dice similarity coefficient (DSC) of 90.5% on a data set of 510 images. However, this method relies greatly on the quality of the saliency maps, which is also not sufficiently robust.

Therefore, how can we have an efficient and robust BUS image segmentation method? We again turned to some excellent classic segmentation methods. It has been reported that tomography watersheds have a certain effect on solving complex segmentation problems and are more stable than other existing methods, but they are sensitive to noise and might cause over-segmentation. In view of this, many scholars have improved watersheds. Huang and Chen [10] combined a watershed with the active contour model to obtain a relatively accurate tumour boundary. In 2009, Gomez used a marked watershed (MW) algorithm incorporating morphological techniques and an average radial derivative function [28]. The method was improved by using an anisotropic diffusion filter guided by texture descriptors derived from a set of Gabor filters and creating segmenting functions generated by Newton filters to facilitate more precise segmentation [29]. However, the anisotropic diffusion filter requires many iterations to obtain a good preprocessing result, which takes a long time. In addition, the acquisition of the marker function is slightly complex, which reduces the efficiency of the algorithm. In view of these two problems, we made improvements in a previous study [30]. We combined contrast-limited adaptive histogram equalization (CLAHE) and curvature filtering to preprocess the images and used a morphological method to obtain the marker function, which is simple and efficient. However, this method not only improves the segmentation accuracy and DSC but also brings a higher false positive rate (FPR), which means that many false positive tissues are also segmented. Therefore, to further improve the performance, this paper makes technical contributions that are summarized as follows:

1. We use CLAHE and a side window filter (SWF) to enhance the lesion contour and eliminate the influence of noise. Compared with some other preprocessing methods, it is the most beneficial to BUS image segmentation. We propose an embedded segmentation method, adaptive morphological snake (AMS). It is more robust and stable than MS when processing complex datasets with different sizes of lesions collected from different types of ultrasound equipment.

2. We propose an optimized marked watershed segmentation method, adaptive morphological snake based on marked watershed (AMSMW). Its marker region is corrected by AMS. Taking full consideration of the advantages of classical segmentation algorithms, such as the level set method [5], morphological snake (MS) [9] and MW [31], we find that AMSMW has higher segmentation precision and is 3–4 times faster than other existing methods.

## Results

### Evaluation metrics

We used both area and contour error metrics, which include the accuracy (Acc), true positive ratio (TPR), false positive ratio (FPR), Jaccard index (JI), Dice similarity coefficient (DSC), area error ratio (AER), Hausdorff error (HE), and mean absolute error (MAE), to evaluate dataset A. The calculation formulas of these indicators are listed below. In addition, we used the Dice coefficient (DC), area-under-curve (AUC), precision (PC), sensitivity (Sen), specificity (Sp), F1-score (F1) and mean-intersection-over-union (M-IOU) to evaluate the proposed method on dataset B. The calculation formulas of these indicators can be found in Zhuang’s paper [22]:

1 $$\begin{aligned}&\text {Acc}=\frac{\left( A_{G} \cap A_{S}\right) \cup \left( \text {A}-\text {A}_{\text {G}} \cup \text {A}_{\text {S}}\right) }{\text {A}} \end{aligned}$$

2 $$\begin{aligned}&\text {TPR}=\frac{\left| A_{G} \cap A_{S}\right| }{\left| A_{G}\right| } \end{aligned}$$

3 $$\begin{aligned}&\text {FPR}=\frac{\left| A_{G} \cup A_{S}-A_{G}\right| }{\left| A_{G}\right| } \end{aligned}$$

4 $$\begin{aligned}&\text {JI}=\frac{\left| A_{G} \cap A_{S}\right| }{\left| A_{G} \cup A_{S}\right| } \end{aligned}$$

5 $$\begin{aligned}&\text {DSC}=\frac{2\left| A_{G} \cap A_{S}\right| }{\left| A_{G}\right| +\left| A_{S}\right| } \end{aligned}$$

6 $$\begin{aligned}&\text {AER}=\frac{\left| A_{G} \cup A_{S}\right| -\left| A_{G} \cap A_{S}\right| }{\left| A_{G}\right| } \end{aligned}$$

7 $$\begin{aligned} \text {HE}\left( C_{G}, C_{S}\right) =\max \left\{ \max _{x \in C_{G}}\left\{ d\left( x, C_{S}\right) \right\} \right\} , \max _{x \in C_{S}}\left\{ d\left( y, C_{G}\right) \right\}  \end{aligned}$$

8 $$\begin{aligned} \text{where}, \, d(z, C)=\min _{k \in C}\{\Vert z-k\Vert \}  \end{aligned}$$

9 $$\begin{aligned}&{\text {MAE}}\left( \text {C}_{S}, \text {C}_{G}\right) =1 / 2\left( \sum _{x \in C_{S}} \frac{d\left( x, C_{G}\right) }{n_{G}}+\sum _{\text {y} \in C_{G}} \frac{d\left( y, C_{S}\right) }{n_{S}}\right) \end{aligned}$$

where $A_{*}$ is the number of pixels in *, *A* is the number of pixels contained in the image, and the subscripts G and S represent the ground truth and segmentation result, respectively. *C* represents the contour of the ROI. z and k are the points in the contour.

Xian has noted how important these metrics are [12]. Large JI values and small AER, HE and MEA values indicate good performance. Supposing that JI is small, when AER, HE, and MAE are large, if TPR and FPR are both large, then the lesion was overestimated. If the TPR and FPR are both small, the lesion was underestimated.

### Experiment details

First, we obtain 100 BUS images and 50 BUS images from dataset A and dataset B respectively as the research object of the research experiment on preprocessing method. And the remaining 500 images in dataset A and the remaining 205 images in dataset B are used as the test set for the comparison experiment. Then, we use Python to implement the algorithm and calculate the evaluation metrics. The parameter setting of the AMSMW method is shown in Table 1. Next, we introduce the process of the three experiments in detail.

**Table 1:** Optimal parameter values of the AMSMW method

Method	Initial point	Radius and iterations
AMSMW	The geometric centre of the RROI	$R=\text {w}-20, \text{ iterations }=250, \text{ if } \min (\text {h}, \text {w})>20, \text{ and } \text {h}>1.5 \text {w}$;
		$$R=\text {w}-20 \text{, } \text{ iterations }=120, \text{ if } \min (\text {h},\text {w})>20, \text{ and } \text {w}/1.5<\text {h}<1.5 \text {w}$$;
		$$R=\text {w}-10 \text{, } \text{ iterations }=250, \text{ if } \min (\text {h},\text {w})<20, \text{ and } \text {h}>1.5 \text {w}$$;
		$$R=\text {w}-10 \text{, } \text{ iterations }=120, \text{ if } \min (\text {h},\text {w})<20, \text{ and } \text {h}<1.5\text {w}$$;

#### Finding the most suitable BUS image preprocessing method

We explored the effect of preprocessing methods on the segmentation results by using four preprocessing schemes: SWF, CLAHE&SWF, CLAHE&CF&SWF, and CLAHE&CF to preprocess the 100 images from dataset A and the 50 images from dataset B. In addition, we set up a control group without preprocessing operations to determine the effectiveness of the preprocessing method on the segmentation result.

#### Comparing AMSMW with other classical image segmentation methods and deep learning methods

First of all, we used the best preprocessing method obtained in the previous step to preprocess the test set. Then, we compared the proposed method with some related segmentation methods and RDAU-NET on the test set of dataset A. Finally, we compared the proposed method with the typical deep learning segmentation method on the test set of dataset B.

In terms of traditional segmentation methods, we implemented some related and classical methods that include level set [5], MS [9], MW [31], and FSMW [30]. For the MS method, we set its initialization position and radius to be the centre of the RROI and 70% of the smallest length and width of the RROI. Moreover, it should be noted that we used the same preprocessing method when performing the comparison experiments except for FSMW.

In terms of deep learning methods, many excellent image segmentation models have been borrowed, improved and used. In [22], several typical deep learning segmentation models were compared on dataset B. The results showed that the RADU-NET model performs best. To make an objective comparison between AMSMW and RADU-NET, we performed the following two experiments. In the first experiment, we used five-fold cross-validation method. First of all, we re-divided the test set of dataset A, according to the ratio of training set:validation set:test set is 6:2:2 and then used the angle transformation method to expand each training set by four times, and then used them to train and test RDAU-NET. Finally, we obtained five segmentation results. In order to achieve a scientific and fair comparison, we tested AMSMW on the five test sets to obtain five results too. The second experiment is that we used AMSMW to conduct a segmentation experiment on the test set of dataset B, and compared the quantitative results with those published in the paper[22].

#### Studying the sensitivity of AMSMW to benign and malignant lesions

Benign and malignant tumours are very different in size, morphology, margins, and internal state, which may greatly affect the algorithm’s performance. If a relationship can be found, it will be of great significance to design a more adaptive BUS image segmentation algorithm. Therefore, we conducted an exploratory experiment on the algorithm’s sensitivity in segmenting benign and malignant lesions. The specific operation was to first group dataset A into 250 benign and 250 malignant groups. Then, a quantitative segmentation experiment was performed.

### Experimental results

#### Combined with CLAHE, SWF can enhance the edge of the lesion and contribute to better BUS segmentation results

**Fig. 1:**
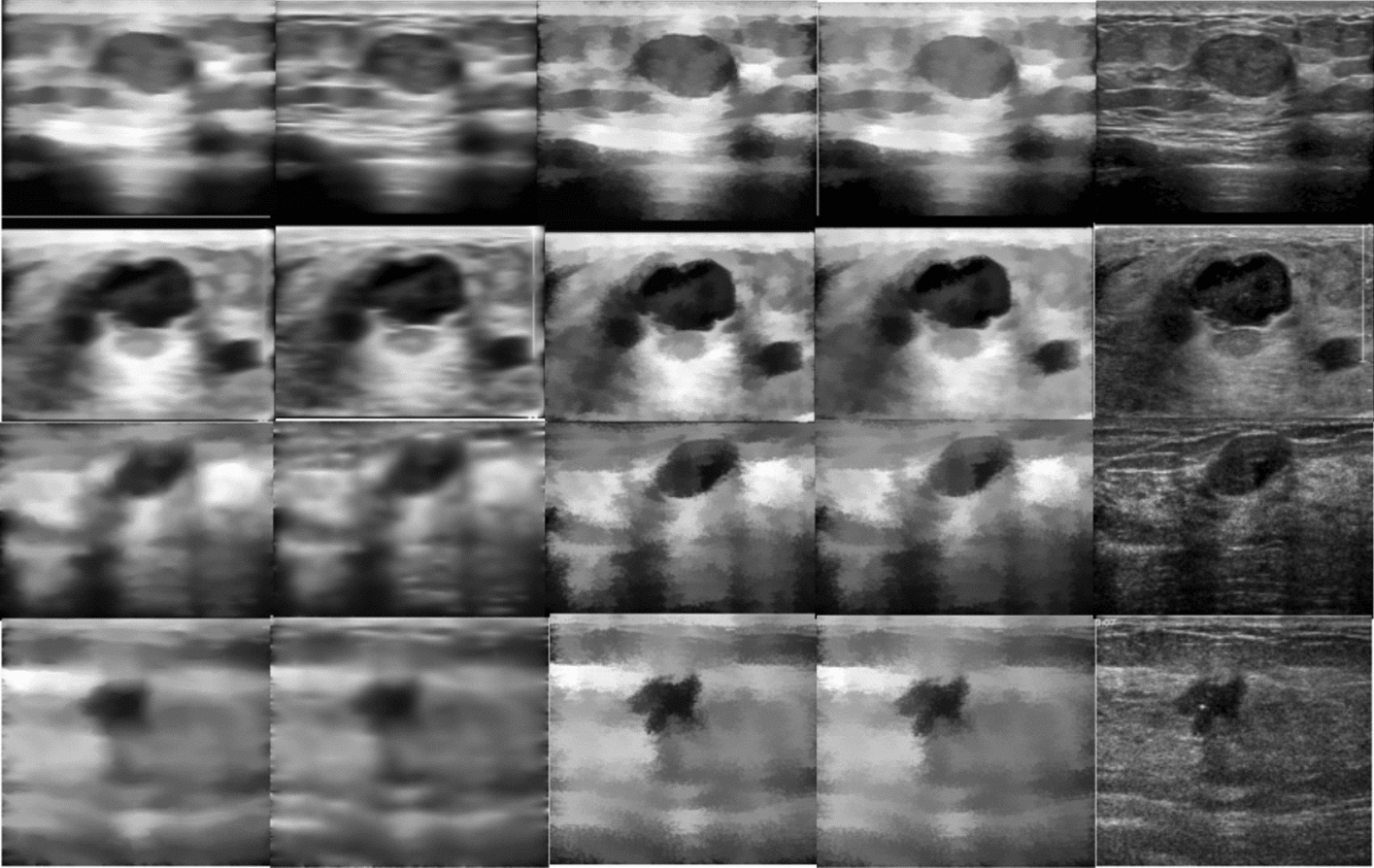
Some examples of the effect of different preprocessing methods. Images in the first row and the second row are from dataset A. And the last two rows are from dataset B. And the corresponding preprocessing schemes for the images from the first column to the last column are contrast limited adaptive histogram equalization+curvature filter+side window filter (CCS), contrast limited adaptive histogram equalization+curvature filter (CC), contrast limited adaptive histogram equalization+side window filter (CS), side window filter (SWF) and none

Quantitative results and some examples of qualitative results are shown in Table 2 and Fig. 1, respectively. As shown in Table 2, the “$\surd$” means “used” and “$\times$” means “not used”. Separate (A) and separate (B) respectively represents the 100 images from dataset A and the 50 images from dataset B used for the preprocessing method experiment. The “Overall” column lists the average values of Dice. Observed form Table 2, we can draw the following three conclusions. First, the segmentation results using preprocessing methods are much better than those without preprocessing methods, and different preprocessing methods improve the segmentation performance to different degrees, which shows that it is very necessary to choose a suitable preprocessing method. Second, the Dice of the CLAHE&SWF method is similar to that of CLAHE&CF&SWF, but the average value of the CLAHE&SWF method is slightly higher, indicating that the method is more stable on different source BUS image datasets. Third, it also shows that CLAHE can improve the contrast and SWF can smooth the noise and well preserve the lesion boundary. It can also be observed directly from Fig. 1. Compared with the images in the last two columns, the contrast of the images in the first three columns on the left which were preprocessed with CLAHE is significantly more balanced and stronger. Images in the third and fourth columns were treated with SWF and their lesion boundaries were clearly highlighted. However, although the images in the first column were also pre-processed by SWF, their lesion boundaries became blurred after using CF.

**Table 2:** Quantitative results of exploring the effect of preprocessing methods on the segmentation results

Methods			Dice (%)		
CLAHE	CF	SWF	Separate(A)	Separate(B)	Overall
$\surd$	$\surd$	$\surd$	$\varvec{87.34}$	86.84	87.09
$\surd$	$\surd$	$\times$	86.18	87.13	86.66
$\surd$	$\times$	$\surd$	87.22	$\varvec{87.52}$	$\varvec{87.37}$
$\times$	$\times$	$\surd$	86.11	86.45	86.28
$\times$	$\times$	$\times$	77.26	75.80	76.53

Bold indicates the best results in the current column

CLAHE: contrast limited adaptive histogram equalization; CF:curvature filter; SWF: side window filter

#### AMSMW performs best on both quantitative results and qualitative results

**Fig. 2:**
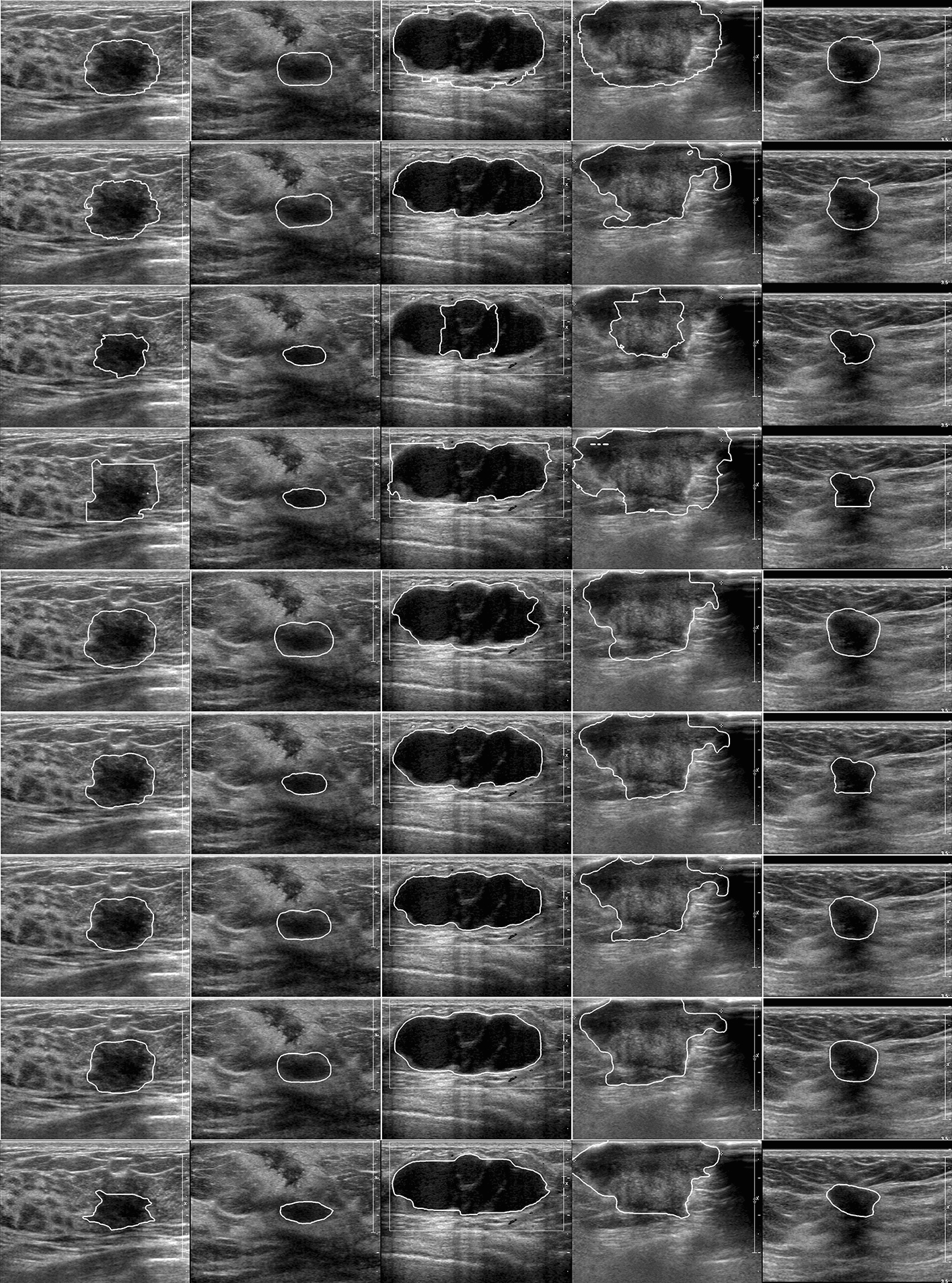
From top to bottom are the qualitative results of marked watershed (MW), level set (level set), morphological snake (MS), adaptive morphological snake (AMS), morphological snake and marked watershed (MS+MW), adaptive morphological snake and marked watershed (AMSMW), level set and marked watershed (level set+MW) and FSMW as well as the ground truth (GT)

First, some relevant and excellent traditional segmentation methods were tested on dataset A, and the quantitative and qualitative results are shown later. It can be observed from Table 3 that even without the preprocessing method, MW still performs the best on TPR, indicating that MW can segment the entire lesion area more sensitively and comprehensively, whereas the error rate is also the highest, causing the FPR to be too high. This means that a large part that does not belong to the lesion area is also segmented, which can be seen intuitively from the qualitative result in Fig. 2. Taking the images in the third and fourth columns as examples, many normal tissues were segmented by MW. However, level set has a lower FPR than MW, indicating that it can effectively improve the ability to identify lesion contours. However, the values of the other indicators of level set were relatively low, indicating that it cannot find the entire lesion area. In addition, it can be seen from the list of standard deviations in Table 3 that the dispersions are generally small, which shows that the data distribution is appropriate and that the experimental results are credible.

**Table 3:** Quantitative results of different segmentation methods

	${ACC \,(\%)}$	${TPR \,(\%)}$	${FPR \,(\%)}$	${DSC\, (\%)}$	${JI \,(\%)}$	${AER\, (\%)}$	$\text {HE}$	$\text {MAE}$
MW	94.37 (±0.03)	**97.64** (±0.02)	79.17 (±0.05)	71.80 (±0.08)	56.68 (±0.10)	81.53 (±0.41)	69.21 (±26.97)	27.88 (±8.81)
level set	94.69 (±0.03)	95.56 (±0.05)	5.24 (±0.03)	69.58 (±0.13)	54.83 (±0.14)	98.11 (±0.78)	72.54 (±26.35)	28.09 (±9.06)
MS	95.76 (±0.04)	63.05 (±0.13)	11.93 (±0.16)	71.57 (±0.10)	56.72 (±0.12)	48.88 (±0.17)	69.91 (±50.21)	22.68 (±13.67)
AMS	94.38 (±0.05)	81.70 (±0.15)	45.32 (±0.28)	72.69 (±0.09)	58.01 (±0.11)	63.62 (±0.32)	72.72 (±44.87)	24.94 (±14.53)
MS+MW	95.92 (±0.03)	67.65 (±0.04)	15.40 (±0.11)	73.57 (±0.12)	59.14 (±0.14)	47.74 (±0.73)	67.00 (±25.24)	21.75 (±7.13)
AMSMW	**96.59** (±0.02)	83.29 (±0.11)	**1.94** (±0.21)	**78.40** (±0.09)	**65.34** (±0.11)	**46.45** (±0.22)	**51.33** (±28.66)	**17.70** (±7.37)
level set+MW	96.25 (±0.03)	91.58 (±0.06)	50.52 (±0.20)	77.47 (±0.09)	64.18 (±0.12)	58.94 (±0.45)	55.85 (±27.01)	19.50 (±7.36)
FSMW	96.19 (±0.03)	92.88 (±0.05)	52.19 (±0.14)	77.53 (±0.09)	64.27 (±0.12)	59.30 (±0.45)	56.08 (±28.61)	19.50 (±7.21)

Bold indicates the best results in the current column

MW: marked watershed [31]; Level set [5]; MS: morphological snake [9]; AMS: adaptive morphological snake; FSMW [30]

Compared with level set, MS has the greatest advantage in that it uses morphological methods to replace the process of solving numerical differential equations, greatly improving the efficiency. Experiments on ordinary notebooks show that it takes 6 s for MS and 17 s for level set to segment an image. At the same time, it can be inferred from the quantitative results that MS is better than level set in most metrics, indicating that it can segment tumours more precisely than level set. However, as shown in Fig. 2, taking the third and fourth columns as examples, there are some lesions with many calcification points inside, which cannot be segmented completely by the MS method. Compared with MS, although AMS is slightly lower than MS on Acc, FPR, AER, HE and MAE, it has obvious advantages on TPR, DSC and JI, showing that AMS is more stable and can obtain more complete lesion. As shown in Fig. 2, images in the fourth row are segmentation results of AMS. It can be observed clearly that although parts of the surrounding normal tissue were mistaken for part of the tumour by AMS, all parts of the tumour were completely included, which is of great significance for AMSMW to obtain a complete marked area later.

By comparing MS+MW with AMSMW, we find that AMS has great effects on improving performance. The level set+MW algorithm is a method in which level set is used instead of AMS as the embedded segmentation method. By comparing the quantitative results of level set+MW with other methods, it can be found that AMSMW has obvious advantages on indicators other than TPR. In addition, as observed from Fig. 2, we find that the qualitative result of AMSMW is much closer to that of GT. Taking the images in the sixth row and fourth column as an example, AMSMW can not only perfectly resist the interference of calcification points and speckle noise in the lesion area, but also resist the interference of back echo of the lesion, so as to accurately identify tumour boundaries. And reduce FPR as much as possible. Moreover, the AMSMW method runs fastest. In summary, we believe that AMSMW has the highest efficiency and effectiveness.

**Fig. 3:**
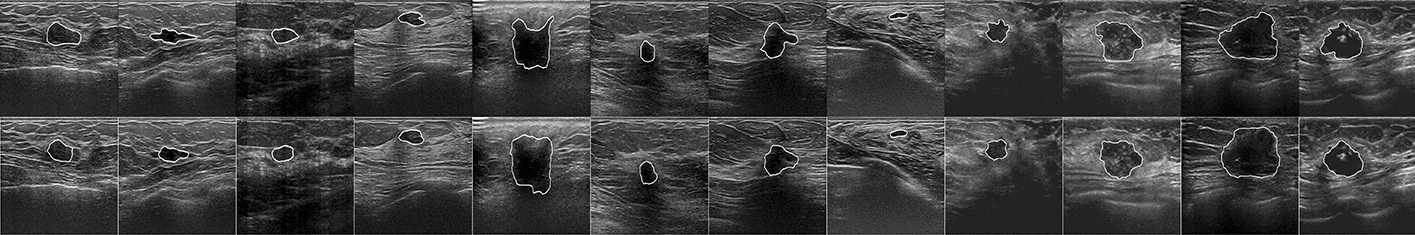
Qualitative segmentation result. The first row is the GT, and the second row is the qualitative result of AMSMW on the shared database. From left to right are image(a), image(b), image(c), image(d), image(e), image(f), image(g), image(h), image(i), image(j), image(k) and image(l). All of them can be found in Figures 11, 12 and 13 of Zhuang’s paper [22], respectively

**Fig. 4:**
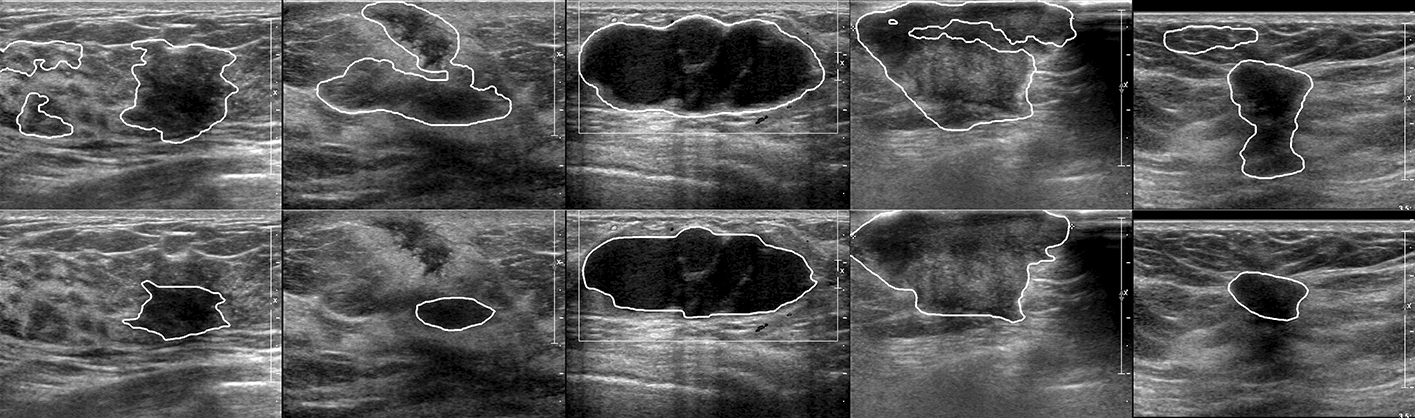
From top to bottom are the qualitative results of RADU-NET and the GT, respectively

Second, we performed segmentation on the 205 images from dataset B, using AMSMW. Furthermore, We performed five-fold cross-validation experiments on the 500 images from dataset A, using RDAU-NET and AMSMW respectively. The quantitative and qualitative results are shown in Table 4 and Fig. 3 and in Table 5 and Fig. 4, respectively. As observed from Table 4, AMSMW is slightly lower than RDAU-NET on SP, PC and M-IOU but obviously higher on the other five metrics. This indicates that AMSMW has good adaptability and can segment lesion areas more precisely than RDAU-NET, which can also be seen more intuitively from Fig. 3. We take images in the first, eighth and tenth column for example. They either have calcification points inside the tumor, severe speckle noise, or strong echoes behind the tumor. Facing with so much interference, AMSMW can still accurately identify tumour boundaries without causing excessive segmentation. RDAU-NET can also find the lesion area, whereas it segments more normal tissue area, increasing the FPR. It can be observed from Fig. 4 clearly, there are relatively more serious problems of over-segmentation and false positive in the segmentation results of RDAU-NET. In addition, observed from Table 5, RDAU-NET does not show a strong generalization ability because it outperforms AMSMW only on Sp and Pc. And it is far worse on the other seven metrics. Overall, it is obvious that if there are not enough training data and excellent hardware resources, even the best deep learning model is not available in regard to new or more complex data. Therefore, there is still a long way for deep learning to go to improve its generalization performance. In other words, although traditional algorithms cannot be fully automated, the semi-automated capability is sufficient to alleviate the burden on doctors, and excellent traditional segmentation algorithms still have good performance when processing complex data.

**Table 4:** Quantitative results of AMSMW on dataset B

	${loss (\%)}$	${Acc (\%)}$	${DC (\%)}$	${Sen (\%)}$	${Sp (\%)}$	${F1 (\%)}$	${Pc (\%)}$	${M-IOU (\%)}$
UNet	17.95	97.57	82.04	84.66	98.91	82.11	81.85	79.83
RDAU	15.30	97.91	84.69	83.19	**99.34**	84.78	**88.58**	**80.67**
AMSMW	**13.75**	**97.96**	**86.25**	**88.79**	98.32	**86.25**	86.26	76.73

Bold indicates the best results in the current column

**Table 5:** Comparison of the quantitative results of rdaunet and AMSMW on the test set of dataset A. fold0, fold1, fold2, fold3 and fold4 are the five test sets in the five-fold cross-validation experiment

		${Loss\, (\%)}$	${Acc\, (\%)}$	${DC\, (\%)}$	${Sen\, (\%)}$	${Sp \,(\%)}$	${F1 \,(\%)}$	${Pc \,(\%)}$	${M-IOU \,(\%)}$
fold0	RDAU	28.22	96.44	71.78	75.43	98.52	74.00	77.96	61.15
	AMSMW	14.55	97.33	85.45	93.64	97.41	85.45	79.89	75.36
fold1	RDAU	25.93	97.17	74.07	73.41	99.07	75.58	83.93	63.65
	AMSMW	14.55	97.26	84.86	92.60	97.33	84.86	79.72	74.42
fold2	RDAU	23.53	96.80	76.49	73.62	99.20	76.47	87.83	66.21
	AMSMW	13.92	97.34	86.08	93.33	97.40	86.08	81.14	76.23
fold3	RDAU	30.57	96.66	69.43	71.52	98.80	69.43	78.85	59.61
	AMSMW	15.15	97.37	84.85	93.21	97.43	84.85	79.66	74.55
fold4	RDAU	23.50	97.14	76.50	78.32	98.77	77.27	80.92	66.23
	AMSMW	15.46	97.35	84.54	92.95	97.55	84.54	79.24	74.20
Average	RDAU	26.35	96.84	73.66	74.46	$\mathbf{98.87}$	74.55	$\mathbf{81.90}$	63.37
	AMSMW	$\mathbf{14.73}$	$\mathbf{97.33}$	$\mathbf{85.16}$	93.15	$\mathbf{97.42}$	$\mathbf{85.16}$	79.93	$\mathbf{74.95}$

Bold indicates the best results in the current column

#### AMSMW is more sensitive to malignant tumours than benign tumours

As shown in Table 6, the performance of AMSMW in segmenting benign and malignant tumours is comparable. From the four indicators, TPR, DSC, JI and AER, we can conclude that AMSMW is more sensitive to discriminating malignant tumours. However, due to the strong echo behind the lesion and the possible strong inner calcification points, AMSMW has a high FPR when segmenting malignant lesions, causing the algorithm to perform poorly on the other four indicators. Therefore, AMSMW is more stable when segmenting benign lesions.

**Table 6:** Quantitative results of the study of the algorithms’ sensitivity in segmenting benign and malignant tumours

	${ACC\, (\%)}$	${TPR\, (\%)}$	${FPR \,(\%)}$	${DSC\, (\%)}$	${JI\, (\%)}$	${AER \,(\%)}$	$\text {HE}$	$\text {MAE}$
B	**97.46**	79.54	**1.94**	76.24	62.63	50.03	**42.15**	**15.71**
M	95.71	**87.03**	3.65	**80.55**	**68.05**	**42.87**	60.51	19.68

Bold indicates the best results in the current column

B: Benign tumour; M: malignant tumour

## Discussion

The proposed method consists of two parts: an image preprocessing scheme and an image segmentation algorithm. These two parts are complementary and inseparable. The goal of the preprocessing scheme is to highlight the boundary of the lesion to obtain more accurate segmentation results. Some preprocessing methods can suppress noise and improve contrast but cannot highlight the boundary. However, some preprocessing methods are the opposite. Therefore, it is necessary to find a suitable image preprocessing scheme. By exploring the effect of preprocessing methods on the segmentation results, we find that for BUS images, CLAHE & SWF is a better image processing method. Theoretically, SWF can preserve boundaries well, while CLAHE can suppress noise and improve contrast. The combination of the two methods is especially suitable for breast cancer ultrasound images with low contrast and speckle noise. In image segmentation, considering the complexity and particularity of BUS images, our goal is to find a robust and efficient lesion segmentation method. MW is very robust in solving complex image segmentation problems. However, its accuracy largely depends on the accuracy of the “marked area”. Theoretically, the “marked area” is the known lesion area. The more accurate the “marked area” is, the higher the accuracy of the algorithm will be. Thus, we mainly optimized MW by improving the method of obtaining the “marked area”. Our idea is to find an excellent algorithm as the acquisition method for the “marked area”. AMS is an improved MS method that can adaptively change the working parameters without tedious calculation of the PED equation. It is proven to be very suitable for acquiring a “marked area” for MW. The experimental results show that the proposed algorithm achieves better segmentation results. Moreover, by comparing it with some other classic traditional segmentation methods, we find that the proposed method is the most efficient and effective algorithm.

In addition, we compared the proposed method with the state-of-the-art deep learning models RDAU-NET and U-NET. It still performed well on most of the metrics on both dataset A and dataset B. Because it does not need a training set, it does not depend too greatly on data sets with different data distributions. Therefore, theoretically, its generalization performance should be better than that of deep learning algorithms. Therefore, in the present era of deep learning, which has attracted much attention and is widely sought after, most deep learning models do not have an ideal generalization ability, which leads to a bottleneck in its continued development. However, the traditional segmentation method is stable and efficient, which provides a way to solve the bottleneck problem of deep learning to a certain extent. Therefore, in future work, we should not neglect classical segmentation methods. Most likely, it would be a good solution to integrate the efficient and stable traditional segmentation method with a deep learning model to complete image segmentation work, and the results would certainly be greatly improved.

By evaluating the sensitivity of the algorithm in segmenting benign and malignant tumours, we find that the proposed method has high sensitivity in the delineation of malignant tumour boundaries and is relatively stable for benign tumours. Therefore, in future work, we could take the strong echo and characteristics of malignant tumours into account and set up an adaptive ideal segmentation method.

Moreover, the current study has great potential for further studies. It will result in better and faster precise diagnosis and treatment of oncological diseases. There are many more medical specialities and diseases [32–34] in which there are known and applicable diagnostic imaging methods, but there are still few predictive modelling bases. Non-invasive diagnostic methods can be used not only in oncology but also in other medical specialities. Therefore, this study can also be applied to computer-aided diagnosis of these diseases.

## Conclusions

In this paper, an efficient semi-automatic BUS image segmentation method was proposed and evaluated quantitatively. It was proven to be the most robust and effective BUS image segmentation method compared with classic traditional segmentation methods and a state-of-the-art deep learning model. In addition, by studying the sensitivity of AMSMW in segmenting benign and malignant lesions, we found that it is more sensitive to malignant lesions and more stable to benign lesions, which is of great significance for algorithm research in precision medicine in the future. Moreover, since the RROI in the proposed method is drawn manually, we are considering adding a deep learning network to automatically identify RROIs and completely liberate radiologists from this task in our future work.

## Methods

**Fig. 5:**
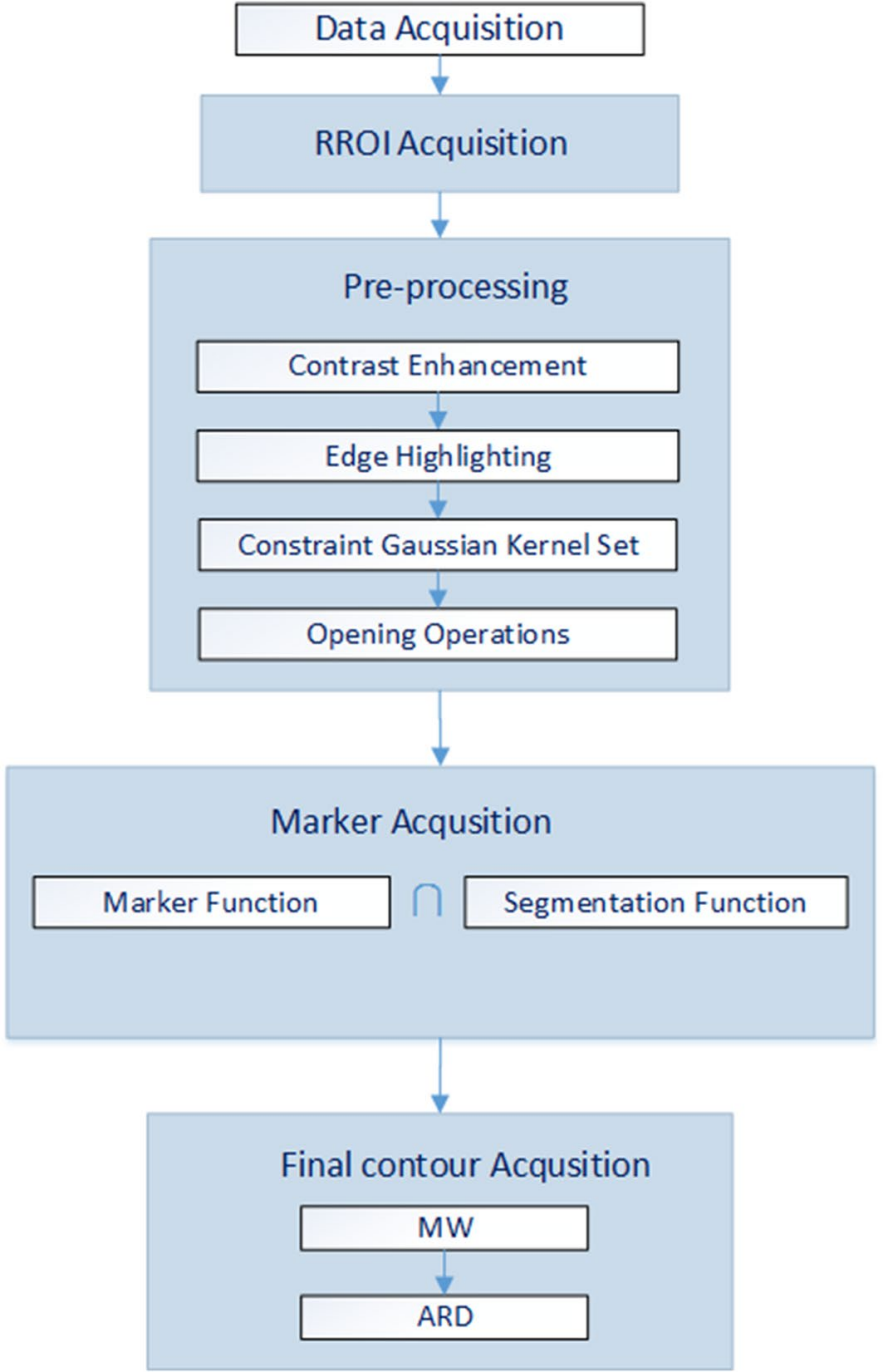
Flowchart of the proposed method

The flowchart of the proposed method is shown in Fig. 5. It consists of five main parts: data acquisition, rectangular region of interest (RROI) acquisition, image preprocessing, marked area acquisition and final contour acquisition. The first three parts are image preparation and preprocessing. The last three parts are the process of image segmentation.

### Data acquisition and difference analysis of the two datasets

Dataset A was collected by us. It has 600 BUS images, which include 300 benign solid cysts and 300 malignant solid cysts. They are captured from different devices, such as GE LOGIQ E9 and PHILIPS EPIQ 5, in a local hospital. The patient information in all images was hidden. An experienced radiologist sketched the lesion boundary for each image as the ground truth (GT). Dataset B is open source. It contains a total of 255 images. Among them, 213 images are from [22], and 42 images are from [35]. To study the generalization ability of the algorithm on different datasets, we analysed the significant differences between dataset A and dataset B. We used a grey level co-occurrence matrix to extract the following statistics for each image: the difference entropy, sum entropy, correlation, angular second moment, sum average, contrast, difference variance, entropy, homogeneity, sum variance, variance and information measure of correlation. Then, we used the Mann-Whitney U test to perform statistical analysis on datasets A and B to obtain the p-value of each statistic, as shown in Table 7. We can see that the p-values of all statistics are less than 0.05. Therefore, we find that datasets A and B have significant differences.

**Table 7:** Analysis of the statistical differences between dataset A and dataset B)

$\text {Textural features}$	${p\, \text{value}}$	$\text {Textural features}$	$p\,\text{value}$
$\text {Difference entropy}$	1.81e−05	$\text {Sum entropy}$	5.45e-27
$\text {Correlation}$	4.31e−08	$\text {Angular second moment}$	5.45e-27
$\text {Sum average}$	0.02	$\text {Contrast}$	2.37e-52
$\text {Variance}$	5.06e−33	$\text {Entropy}$	1.85e-10
$\text {Difference variance }$	7.80e−31	$\text {Homogeneity}$	1.39e-25
$\text {Sum variance}$	1.81e−05	$\text {Variance}$	1.38e-10
$\text {Information measure of correlation}$	7.19e−36		

### RROI acquisition

The RROI was obtained manually by the following steps: first, a point was selected as a starting point by left-clicking the mouse and holding down the left mouse button to move diagonally until the end position was found. Here, we define the RROI’s four vertices as $w_{1}$,$w_{2}$, $h_{1}$, and $h_{2}$, where w and h represent points along the tumour’s width and height, respectively, and the sub-indices 1 and 2 represent the lower and upper limits of the tumour’s width and height, respectively. The geometric centre of the RROI, which will be used later, can be defined as

10 $$\begin{aligned} \hat{\mu }=\left( \mu _{w}, \mu _{k}\right) =\left( w_{1}+\frac{w_{2}-w_{1}}{2}, h_{1}+\frac{h_{2}-h_{1}}{2}\right) . \end{aligned}$$

### Image preprocessing

#### Contrast enhancement

BUS images are characterized by low contrast and considerable noise, which can be improved by applying CLAHE, an optimization method based on adaptive histogram equalization (AHE) that limits the increase in contrast. It effectively overcomes the problem of over-amplifying noise in the AHE algorithm.

#### Edge highlighting

Local windows, whose centres align with the pixels being processed, usually cause blurred edges. To avoid this, [36] proposed SWF, which can significantly preserve edges. Thus, we introduced SWF to highlight the edges of lesions in BUS images. We give a brief introduction to SWF, and more information can be found in [36].

**Fig. 6:**
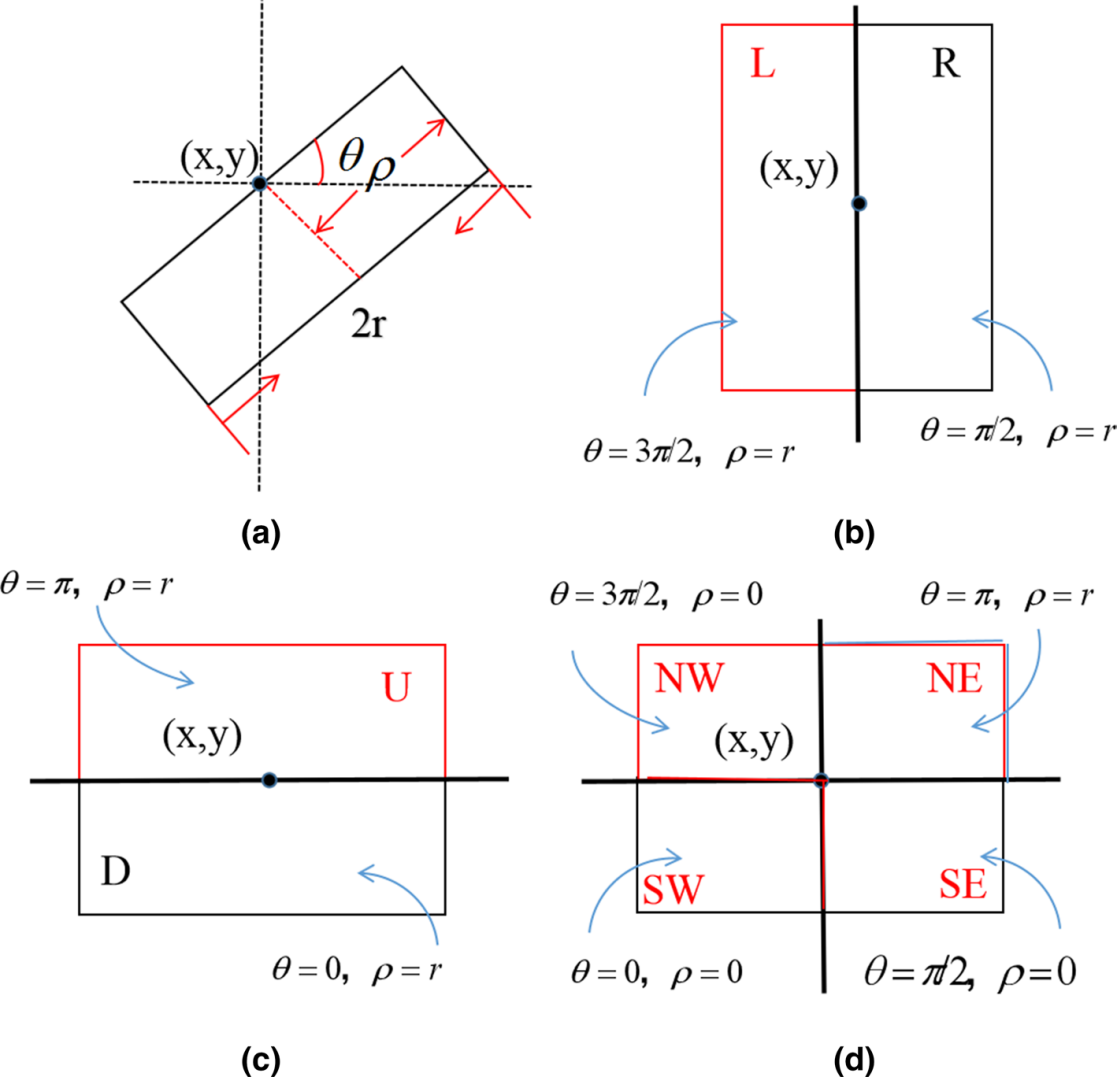
Definition of side windows. *r* is the radius of the window. **a** Side window in the continuous case. **b** The left (L) and right (R) side windows. **c** The up (U) and down (D) side windows. **d** The northeast (NE), northwest (NW), southeast (SE) and southwest (SW) side windows

As shown in Fig. 6, eight side windows are defined only in a discrete case, where (x, y) are the coordinates of target pixel i and r and $\theta$ are the radius and angle of the window, respectively. $\rho \in \{\hbox {o},\hbox {r}\}$ , $\theta =\hbox {k}\times \pi /2$, and k$\in$[0,3]. Thus, we can obtain four side windows, $W_{Di}$, $W_{Ri}$, $W_{Ui}$ and $W_{Li}$, by setting $\rho$=r, which aligns i with their sides. While $\rho$=0, we have $W_{SWi}$, $W_{SEi}$, $W_{NEi}$ and $W_{NWi}$, which align i with their corners. For each pixel, the process of filtering can be regarded as the process of finding the $I_{am}$ value, which satisfies

11 $$\begin{aligned} \begin{array}{l} I_{\text {m}}=\arg \min _{n \in S}\left\| q_{i}-I_{n}\right\| _{2}^{2} \\ \end{array} \end{aligned}$$

where,

12 $$\begin{aligned} \text {I}_{n}=\frac{1}{N_{n}} \sum _{j \in w_{i}^{n}} w_{i j} q_{j} \end{aligned}$$

13 $$\begin{aligned} \text {N}_{n}=\sum _{j \in w_{i}^{n}} w_{i j}, n \in S  \end{aligned}$$

$W_{ij}$ is the weight of pixel j, which neighbours pixel i, based on the filtering kernel F; $q_{j}$ is the intensity of image q at location i; and S=L, R, U, D, NW, NE, SW, SE is the set of side window indices. The result of filtering by SWF is defined as

14 $$\begin{aligned} \text {I}_{\text {SWF}}^{\prime }=\arg \min _{\forall I_{i}^{\theta , \rho , r}}\left\| q_{i}-I_{i}^{\prime \theta , \rho , r} \right\| ^{2}_2, \end{aligned}$$

where

15 $$\begin{aligned} \text {I}_{i}^{\prime \theta , \rho , r}=F\left( q_{i}, \theta , \rho , r\right) . \end{aligned}$$

$I_{i}^{'\theta ,\rho ,\gamma }$ is the result for the eight side windows when $\rho \in \{\hbox {o},\hbox {r}\}$, $\theta =\hbox {k}\times \pi /2$, and k$\in$[0,3].

#### Constrained Gaussian kernel set

Similar to the method proposed in [30], we multiply Gaussian functions with the filtered image $I_{CF}$ to obtain the region of interest (ROI). However, the difference is that we use a union of five constrained Gaussian distributions that have the same variances to make the lesion area more prominent:

16 $$\begin{aligned} \sigma _{w}=\frac{w_{2}-w_{1}}{2},\sigma _{h}=\frac{h_{2}-h_{1}}{2} \end{aligned}$$

The only difference between the five constrained Gaussian functions is the centre position. One is centred at the geometric centre of the RROI, and the other four are translated by half of the diagonal lengths in the four diagonal directions of the RROI. Hence, taking the Gaussian function centred at the geometric centre of the RROI as an example, its function can be expressed as

17 $$\begin{aligned} G(m, n)=\frac{\exp \left( -1 /2 \left( \frac{(\hat{p}-\hat{\mu })^{2}}{\sigma _{w}^{2}}+\frac{(\hat{p}-\hat{\mu })^{2}}{\sigma _{h}^{2}}\right) \right) }{2 \pi \sqrt{{\text {det}} s_{\sigma }}}, \end{aligned}$$

where $\hat{p}$(m,n) represents the pixel’s location and $s_{\alpha }$ is the diagonal covariance matrix. This can be expressed as

18 $$\begin{aligned} s_{\sigma }=\left( \begin{array}{l} \sigma _{w}^{2}\quad 0 \\ 0 \quad \sigma _{h}^{2} \end{array}\right) \end{aligned}$$

We superimpose these five Gaussian functions to obtain their union $G_{T}$ and then multiply it with $I_{CF}$, which was negative previously:

19 $$\begin{aligned} J(m, n)=G_{T}(m, n)\cdot \left( 1-\frac{I_{C F}(m, n)}{\max _{\hat{p}}\left( I_{C F}(m, n)\right) }\right) \end{aligned}$$

**Fig. 7:**
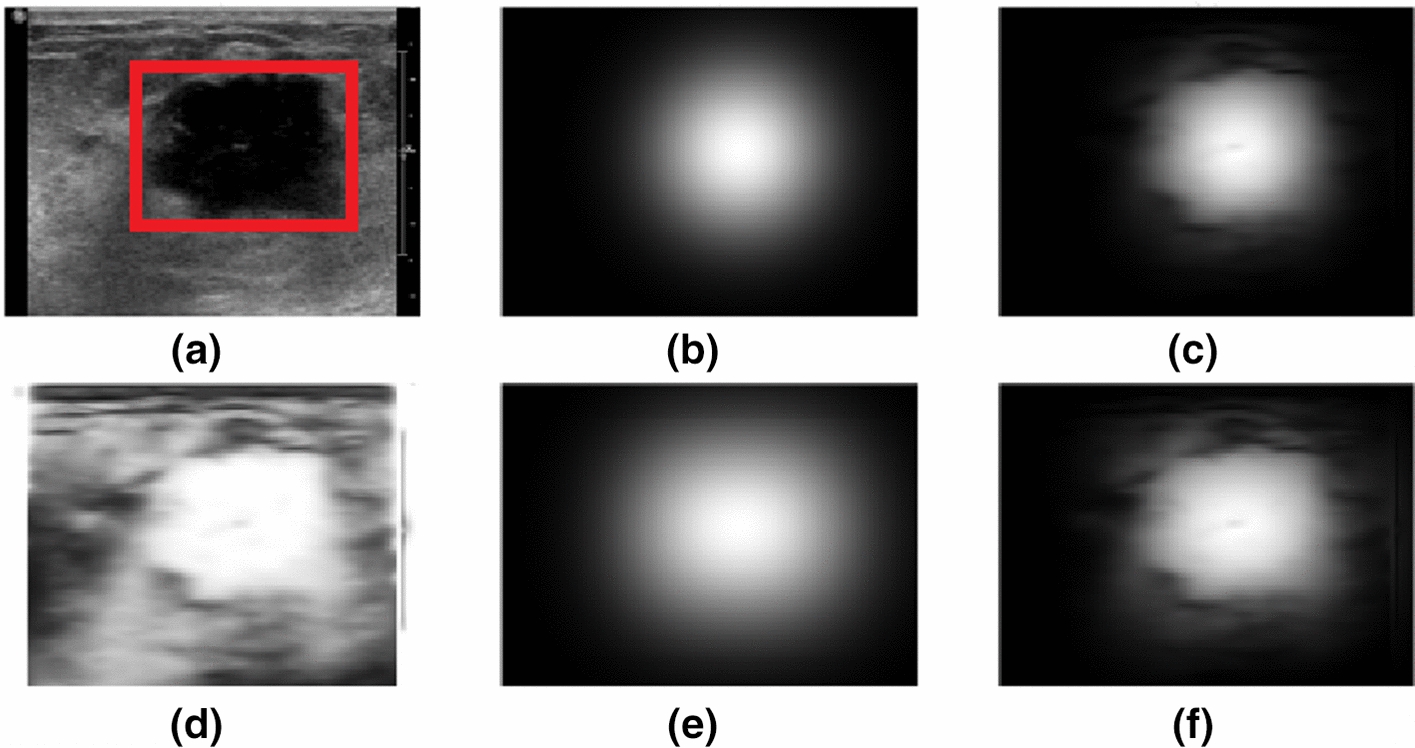
Process of obtaining the region of interest (ROI). **a** Original image with the rectangular region of interest (RROI) drawn by hand; **b** a constrained Gaussian function centred at the geometric centre of the RROI; **c** the resulting image after multiplying (**b**) and (**d**); **d** the negative of $I_{CF}$; **e** the union of five constrained Gaussian functions; **f** the resulting image, which is denoted as J, after multiplying (**e**) and (**d**)

Therefore, a specific highlighted ROI, whose surrounding tissue is greatly darkened, is obtained, as shown in Fig. 7. The experiments show that the illuminated ROIs obtained by these five Gaussian function sets are more complete than before, which is significant for determining accurate tumour boundaries and performing efficient segmentation.

Then, the input image of the marker function, JmnM, and the input image of the segmentation function, JmnN, are obtained separately by performing the opening operation on J(m,n) using a 9- and a 15-pixel radius disk, respectively.

### Marked area acquisition

The MW algorithm depends greatly on the marked area. The proposed method mainly improves the method of obtaining the marked area. We obtain the marked area by taking the intersection of the marker function and segmentation function.

#### Marker function

Similar to [30], we obtain the marker function through a series of morphological operations. First, we binarize JmnM with 1-255 as the threshold. The 255 binarized images are denoted as $f_{p}^{th}$(m,n), (th=1,2,...,255), corresponding to 255 marker functions. Referring to Eq. 20, the marker function can be obtained by performing morphology operations:

20 $$\begin{aligned} f_{Mar}^{th}(m, n)=f_{ext}^{th}(m, n) \cup f_{int}^{th}(m, n) \end{aligned}$$

21 $$\begin{aligned} f_{ext}^{th}(m, n)=\delta _{B_{2}}\left( \delta _{B_{1}}\left( f_{p}^{th}(m, n)\right) \right) -\varepsilon _{B_{2}}\left( \delta _{B_{1}}\left( f_{p}^{th}(m, n)\right) \right) \end{aligned}$$

22 $$\begin{aligned} f_{int}^{th}=\varepsilon _{B_{1}}\left( f_{p}^{th}(m, n)\right)  \end{aligned}$$

where $f_{p}^{th}$ represents the marker function and $f_{ex}^{th}$ and $f_{in}^{th}$ represent the external and internal markers, respectively. $\delta$ and $\varepsilon$ are morphological dilation and erosion, respectively, and $B_{1}$ and $B_{2}$ are two structural elements with a 15-pixel-radius disk and a 15-pixel-wide square, respectively.

#### Segmentation function

In [30], we discussed and proved that the segmentation function plays a large role in whether we can obtain accurate markers and makes a great contribution to obtaining good segmentation results. Therefore, to obtain more precise segmentation results, we evaluate the existing segmentation methods and propose an optimized method to obtain the segmentation function.

(1) MS: Let $u_{e}$: $R^{+} \times R^{2} \rightarrow$R be an implicit representation of C such that C(t)=(x,y); u(t,(x,y))=0. MS uses a combination of binary morphological operators whose infinitesimal behaviour is equivalent to the flow expressed by the active contour PDE. Therefore, the curve is given as the zero level set of a binary piecewise constant function u: $R^{2}$ $\rightarrow$ $\{$0,1$\}$. We take u(x)=1 for every point x inside the curve and u(x)=0 for every point x outside the curve. The morphological operators act on u and implicitly evolve the curve:

23 $$\begin{aligned} \frac{\partial u}{\partial t}=g(I)|\nabla u|\left( div\left( \frac{\nabla u}{|\nabla u|}\right) +v\right) +\nabla g(I) \nabla u, \end{aligned}$$

where v$\in$ R is the balloon force parameter and g(I) selects which regions of I attract the curve. In the MS model, we use two common morphological operators: erosion and dilation. The dilation of a function is defined as

24 $$\begin{aligned} \left( D_{h} u\right) (\text {x})=\sup _{y \in h B} u(\text {x}-\text {y}). \end{aligned}$$

The erosion is defined as

25 $$\begin{aligned} \left( E_{h} u\right) (\text {x})=\inf _{y \in h B} u(\text {x}-\text {y}). \end{aligned}$$

The balloon force PDE can be expressed as

26 $$\begin{aligned} \frac{\partial u_{ball}}{\partial t}=g(I) \cdot V \cdot \left| \nabla u_{ball}\right| . \end{aligned}$$

Given that the snake evolution at iteration n is $u^{n}$: $R^{2}$ $\rightarrow$ $\{$0,1$\}$, it can be solved using the following morphological approach:

27 $$\begin{aligned} u^{n+1}\left( x_{i}\right) =\left\{ \begin{array}{ll} \left( D_{d} u^{n}\right) \left( x_{i}\right) &{} \text{ if } g(I)\left( x_{i}\right)>\theta \text{ and } v>0 \\ \left( E_{d} u^{n}\right) \left( x_{i}\right) &{} \text{ if } g(I)\left( x_{i}\right) >\theta \text{ and } v<0 \\ u^{n}\left( x_{i}\right) &{} \text{ otherwise } \end{array}\right. \end{aligned}$$

where $D_{d}$ and $E_{d}$ are the discrete versions of dilation and erosion. Therefore, the morphological implementation of Eq. 27 can be expressed as

28 $$\begin{aligned}&u^{n+\frac{1}{3}}(x)=\left\{ \begin{array}{ll} \left( D_{d} u^{n}\right) \left( x_{i}\right) &{} \text{ if } |v| g(I)\left( x_{i}\right)>\theta \text{ and } v>0 \\ \left( E_{d} u^{n}\right) \left( x_{i}\right) &{} \text{ if } |v| g(I)\left( x_{i}\right) >\theta \text{ and } v<0 \\ u^{n}\left( x_{i}\right) &{} \text{ otherwise } \end{array}\right. \end{aligned}$$

29 $$\begin{aligned}&u^{n+\frac{2}{3}}\left( x_{i}\right) =\left\{ \begin{array}{l} 1 \quad \text{ if } \nabla u^{n+\frac{1}{3}} \nabla g(I)\left( x_{i}\right) >0 \\ 0 \quad \text{ if } \nabla u^{n+\frac{1}{3}} \nabla g(I)\left( x_{i}\right) <0 \\ u^{n+\frac{1}{3}} \quad \text{ if } \nabla u^{n+\frac{1}{3}} \nabla g(I)\left( x_{i}\right) =0 \\ \end{array}\right. \end{aligned}$$

30 $$\begin{aligned}&u^{n+1}\left( x_{i}\right) =\left\{ \begin{array}{lll} S I_{d} \circ I S_{d} u^{n+\frac{2}{3}}\left( x_{i}\right) &{} \text{ if } \quad g(I)(x)>0 \\ u^{n+\frac{2}{3}}(x) &{} \text{ otherwise } \end{array}\right. \end{aligned}$$

where $SI_{d}$ and $IS_{d}$ are smoothing operators. In a binary image u, $SI_{d}$ works only on white pixels, and $IS_{d}$ works only on black pixels. Taking $SI_{d}$ as an example, for every white pixel $x_{1}$ in a binary image, the $SI_{d}$ operator looks for small (3-pixel-long) straight lines of white pixels that contain $x_{1}$. This search is done in the four possible orientations corresponding to the four segments in P, where P is a collection of four discretized segments centred at the origin:

31 $$\begin{aligned} P=\left\{ \begin{array}{lll} \{(0,0), &{} (1,0), &{} (-1,0)\}, \\ \{(0,0), &{} (1,1), &{} (0,-1)\}, \\ \{(0,0), &{} (0,1), &{} (-1,-1)\}, \\ \{(0,0), &{} (1,-1), &{} (-1,1)\} \end{array}\right\} \end{aligned}$$

**Fig. 8:**
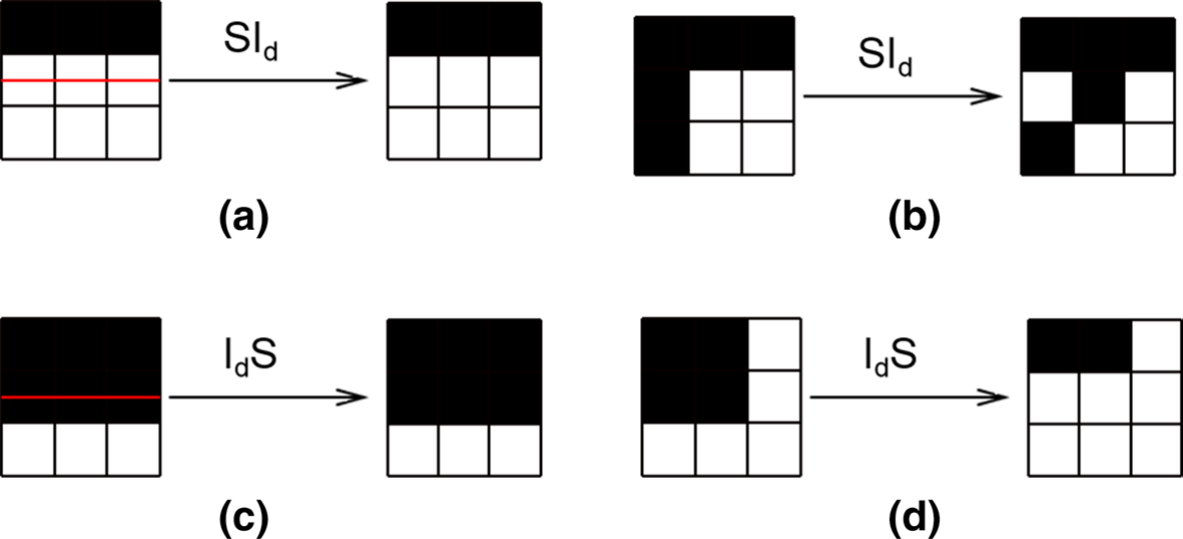
Some examples of the effect of the $SI_{d}$ and $Id_{S}$ operators. They retain the points where a straight line (marked in red) is found, as shown in **a** and **c**. However, when the centre point is not on any straight line, it will be changed, as shown in **b** and **d**

If no straight line exists, the pixel is made black (see Fig. 8). Sharp edges (Fig. 8b and d) are detected and removed as pixels that are not part of a straight line. White pixels on smooth edges (Fig. 8a and c) remain unchanged.

If no straight line exists, the pixel is made black (see Fig. 8). Sharp edges (Fig. 8b and d) are detected and removed as pixels that are not part of a straight line. White pixels on smooth edges (Fig. 8a and c) remain unchanged.

(2) AMS: Considering the different sizes of tumours, MS is not sensitive to especially large or small tumours. Therefore, we propose AMS, which is an optimized model based on the MS model, by applying adjustments to choose appropriate parameters.

In the AMS model, different shapes and types of tumour are considered. We use the geometric centre of the manually acquired RROI as the initial point and adjust the radius and iterations of the circle level set in real time according to the aspect ratio of the tumour. In the MS model, these parameters are fixed. Table 8 lists the relevant adjustable parameters of MS and AMS.

**Table 8:** Adjustable parameters of morphological snake (MS) and adaptive morphological snake (AMS)

Method	Initial point	Radius	Iterations
MS	A fixed point (the default is the centre of the image)	A fixed value (the default is 75% of the smallest image dimension)	A fixed value
AMS	The geometric centre of the RROI	Real-time adjusted value	Real-time adjusted value

Finally, we can find the minimum boundary $f_{sm}th(m, n)$, referring to Eq. 32. Then, we obtain the marked area by performing a closing operation with a 25-pixel-radius disk after binarization:

32 $$\begin{aligned} f_{s m}^{t h}(m, n)=f_{s e g}(m, n) \cap f_{m a r}^{t h}(m, n) \end{aligned}$$

### Final contour acquisition

First, we obtain 255 candidate contours by setting $f_{label}^{th}$(m, n) as the input of MW, referring to (33).

33 $$\begin{aligned} f_{\text {MW}}^{t h}(m, n)=\text {MW}\left( f_{\text{ label }}^{t h}(m, n)\right) \end{aligned}$$

Second, we take the contour corresponding to the largest average radial derivative (ARD) value as the final contour. After the ARD is calculated for some sample images, 96 is determined as the average threshold value corresponding to the maximum ARD (for more details, please refer to [30]). To improve the efficiency of the algorithm and ensure that the selected boundary line is close to the ideal boundary line, we directly take the candidate boundary corresponding to the threshold of 96 as the final contour, thus avoiding the calculation of the ARD for 255 candidate boundaries in an image.

## Data Availability

The datasets analysed during the current study are available from the corresponding author on reasonable request.

## Abbreviations

BUS: Breast ultrasound
CLAHE: Contrast limited adaptive histogram equalization
SWF: Side window filter
RROI: Rectangular region of interest
AMS: Adaptive morphological snake
MW: Marked watershed
